# Prion Shedding from Olfactory Neurons into Nasal Secretions

**DOI:** 10.1371/journal.ppat.1000837

**Published:** 2010-04-15

**Authors:** Richard A. Bessen, Harold Shearin, Scott Martinka, Ryan Boharski, Diana Lowe, Jason M. Wilham, Byron Caughey, James A. Wiley

**Affiliations:** 1 Veterinary Molecular Biology, Montana State University, Bozeman, Montana, United States of America; 2 Laboratory of Persistent Viral Diseases, National Institute of Allergies and Infectious Diseases, Hamilton, Montana, United States of America; University of Edinburgh, United Kingdom

## Abstract

This study investigated the role of prion infection of the olfactory mucosa in the shedding of prion infectivity into nasal secretions. Prion infection with the HY strain of the transmissible mink encephalopathy (TME) agent resulted in a prominent infection of the olfactory bulb and the olfactory sensory epithelium including the olfactory receptor neurons (ORNs) and vomeronasal receptor neurons (VRNs), whose axons comprise the two olfactory cranial nerves. A distinct glycoform of the disease-specific isoform of the prion protein, PrP^Sc^, was found in the olfactory mucosa compared to the olfactory bulb, but the total amount of HY TME infectivity in the nasal turbinates was within 100-fold of the titer in the olfactory bulb. PrP^Sc^ co-localized with olfactory marker protein in the soma and dendrites of ORNs and VRNs and also with adenylyl cyclase III, which is present in the sensory cilia of ORNs that project into the lumen of the nasal airway. Nasal lavages from HY TME-infected hamsters contained prion titers as high as 10^3.9^ median lethal doses per ml, which would be up to 500-fold more infectious in undiluted nasal fluids. These findings were confirmed using the rapid PrP^Sc^ amplification QuIC assay, indicating that nasal swabs have the potential to be used for prion diagnostics. These studies demonstrate that prion infection in the olfactory epithelium is likely due to retrograde spread from the olfactory bulb along the olfactory and vomeronasal axons to the soma, dendrites, and cilia of these peripheral neurons. Since prions can replicate to high levels in neurons, we propose that ORNs can release prion infectivity into nasal fluids. The continual turnover and replacement of mature ORNs throughout the adult lifespan may also contribute to prion shedding from the nasal passage and could play a role in transmission of natural prion diseases in domestic and free-ranging ruminants.

## Author Summary

Prion diseases are fatal neurodegenerative diseases and in ruminants they can be highly contagious, yet the route of transmission among sheep with scrapie or deer with chronic wasting disease is not completely understood. In this study, we examined the hypothesis that prion infection in peripheral neurons that are located at a mucosal surface can result in the release of prion infectivity into bodily secretions. Our findings indicate that prion infection of the olfactory system leads to a high level of infection of olfactory neurons in the sensory epithelium of the nasal cavity, likely by retrograde spread in the olfactory nerve. Prions were also located in the sensory cilia of olfactory neurons and since these structures project into the airway of the nasal cavity, we were able to detect moderate levels of prion infectivity in nasal secretions. These findings demonstrate how prions can disseminate within a host to a peripheral neuron at the mucosa and subsequently release infectivity into bodily fluids. Furthermore, olfactory sensory neurons undergo a continual turnover throughout the adult lifespan and the loss of prion-infected neurons at the olfactory mucosa could also result in continuous shedding of prion infectivity and serve as a mechanism for disease transmission.

## Introduction

Recent evidence indicates that natural prion diseases such as scrapie in sheep and chronic wasting disease in cervids, which cause a progressive fatal neurodegeneration, can be highly contagious [1], [2]. In a large scale study of hunter harvested cervids in the 1990's, the Colorado Division of Wildlife reported a CWD infection rate of 4.9% in mule deer, 2.1% in white tailed deer, and 0.5% in elk [3]. An even higher prevalence rate for CWD infection is found in the core area of infection in south central Wisconsin where the prevalence of CWD in adult buck white-tail deer was 15.5% in 2008 [4]. In cervid game farms the prevalence of CWD infection can vary widely, but has been reported as high as 83% in white-tail deer and 67% in captive mule deer [1], [2]. Similarly, in domestic sheep there is a genetic predisposition for scrapie among certain breeds [5]–[8] and the annual prevalence of disease can range from less than one percent to greater than 20% in adults [9], [10]. In a recent report, the culling and testing of two endemically infected flocks revealed that 58% of sheep were positive for the abnormal isoform of the prion protein, PrP^Sc^, despite a very low number of clinical scrapie cases [11]. These infection rates indicate that a relatively high percent of certain sheep breeds and North American deer in endemic areas can have a subclinical prion infection, but that typically only a small percent of animals exhibit symptoms of disease at a given time. These studies also suggest that these prion diseases have the potential to be highly contagious under certain conditions, which are not completely understood (e.g., animal density, number of contacts, prion protein genotype, etc). Despite these recents developments in identifying a high prevalence of infection for scrapie and CWD in domestic and free-ranging ruminants, the mode(s) of prion transmission among hosts is not well understood.

Recent evidence indicates that scrapie can be transmitted via milk from an infected ewe to lambs from a scrapie-negative flock, possibly via infected macrophages [12], [13]. Other studies have found scrapie infection in placenta from infected ewes and although infection does not appear to transmit in utero, after birthing, placenta could lead to infection of newborn lambs or contaminate the environment for subsequent horizontal transmission [14], [15]. These sources of prion infectivity have not been confirmed in CWD infected cervids, but in both prion diseases, blood does contain prion infectivity [16], [17]. However, blood borne prion transmission via vectors or other mechanisms has not been reported in ruminants. Recent studies demonstrate the presence of prion infectivity in both saliva and urine of CWD infected deer or rodent prion models, and successful transmission of CWD to deer has been achieved following experimental oral ingestion of saliva obtained from infected deer [17], [18]. Feces from CWD-infected deer have also been demonstrated to contain prion infectivity, although the amount of infectious agent was extremely low [19]. It has not been resolved whether infection from these fluids and tissues can directly or indirectly transmit prion infection in natural settings, but environmental contaminants are also likely sources for prion transmission [20]. In addition to exposure to prion agent, the dose of agent exposure is likely to be a primary factor in determining disease transmission. The reported amount of prion infectivity in saliva, urine, milk, and feces from prion-infected animals is very low [17]–[19], [21].

Since neurons are a principal target of prion infection and the brain has the highest amount of prion infectivity, we propose that peripheral neurons located at mucosal surfaces can replicate prions to high levels and release prion infectivity into mucosal fluids. The olfactory mucosa has previously been demonstrated to be a site of prion infection in animals and humans [22], [23] and it contains olfactory receptor neurons, which are a population of neurons that continue to turnover and replenish during adult life. Prior studies have demonstrated that the olfactory epithelium, vomeronasal epithelium, nerve bundles, and/or nasal associated lymphoid tissue is a target for prion infection in humans with classical Creutzfeldt-Jakob disease, sheep scrapie, deer and elk with CWD, or in rodent models of prion infection [22]–[28]. In the present study we investigated the cell type and location of prion infection in the olfactory mucosa of hamsters infected with the HY strain of transmissible mink encephalopathy (TME) [29] as well as the amount of prion infectivity in the olfactory mucosa and in nasal fluids. Our findings indicate that olfactory or vomeronasal receptor neurons are the principal, and perhaps only, site of prion infection in the sensory epithelium. The olfactory mucosa contained high amounts of prion infectivity while nasal fluids had low-to-moderate levels. The presence of prion infection in the cilia of olfactory neurons suggests that prion infectivity can be released into the lumen of the nasal airway, possibly during the normal and frequent turnover of these neurons. Our studies suggest that prion infection of olfactory receptor neurons can result in shedding of prion infectivity into nasal fluids, which may contribute to prion transmission to naïve hosts.

## Results

### Prion infection of olfactory system

**Figure 1:**
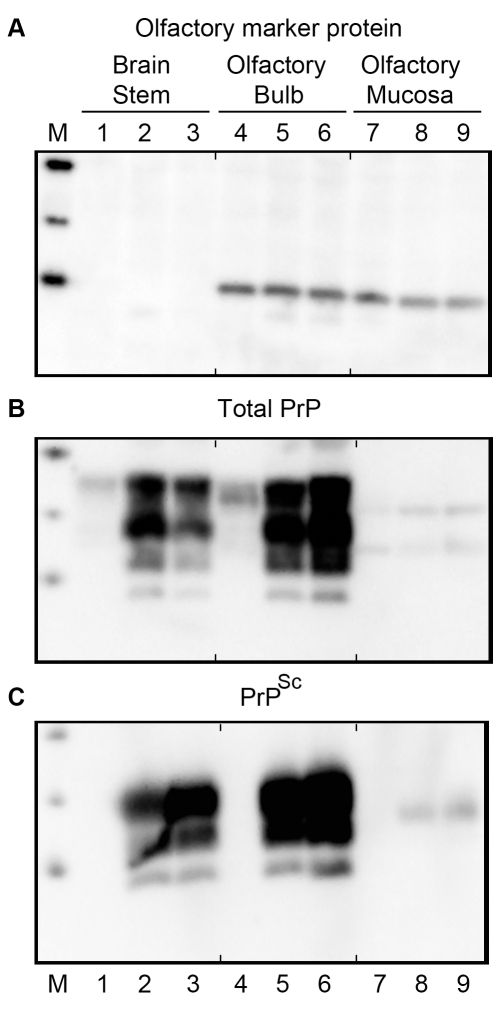
Western blot for olfactory marker protein and prion protein in brainstem, olfactory bulb, and olfactory mucosa following HY TME infection of hamsters. Tissue from age-matched, mock-infected (lanes 1, 4, 7) and HY TME-infected (lanes 2, 3, 5, 6, 8, 9) hamsters following intracerebral (lanes 2, 5, 8), and intra-olfactory bulb (lanes 1, 3, 4, 6, 7, 9) inoculation were homogenized and 50 (*A*, *B*) or 100 (*C*) micrograms of protein was analyzed per lane. In *C*, protein samples were digested with 10 ug/ml of proteinase K for 37°C for 1 hour prior to SDS-PAGE. This treatment removes PrP^C^ in mock-infected samples and truncates PrP^Sc^ in HY TME lysates. Total prion (PrP) protein (*B*) and PrP^Sc^ (*C*) was immunodetected with anti-PrP 3F4 monoclonal antibody, while olfactory marker protein (*A*) was immunodetected with polyclonal goat serum to OMP. OMP is present in both the olfactory bulb, which contains the olfactory nerve and terminals, and in the olfactory mucosa that contain the soma, dendrites, and axons of ORNs. Marker (M) polypeptides correspond to 20, 30, and 40 kDa. The short vertical tick marks at the top and bottom of each panel are placed before lanes 4 and 7 in order to align lanes between each panel.

To test for the presence of olfactory receptor neurons (ORNs) in the olfactory bulb and olfactory mucosa of Syrian golden hamsters, tissue lysates from the cerebellum-brainstem, olfactory bulb, and olfactory mucosa after extraction from the nasal turbinates were assayed for olfactory marker protein (OMP) by western blot (Figure 1A). Both the olfactory bulb and olfactory mucosa had comparable levels of OMP (a 19 kilodalton protein) on a per protein basis, but the cerebellum-brainstem homogenate, which does not contain ORNs, did not have evidence of OMP immunoreactivity (Figure 1A, lanes 1, 4 and 7). To investigate prion infection of the olfactory system, initially the level of PrP^C^ was measured in these same tissues. A western blot for PrP^C^ in tissue homogenates from age-matched, normal mock-infected hamsters revealed the typical pattern of PrP^C^ polypeptides, whose major polypeptides range from 33 to 35 kilodalton (kDa), in lysates from the cerebellum-brainstem and olfactory bulb (Figure 1B, lanes 1, 4). The amount of PrP^C^ in these parts of the brain was four to eight times greater than in the olfactory mucosa (Figure S1). In the olfactory mucosa, the typical brain-associated PrP^C^ polypeptides were not found, but two major immunoreactive anti-PrP 3F4 polypeptides, at approximately 25 and 31 kDa, were present (Figure 1B, lane 7). Enzymatic deglycosylation of PrP^C^ in olfactory bulb and nasal turbinate lysates produced a single immunoreactive polypeptide at approximately 27.8 kDa, indicating that the differences in PrP^C^ molecular weight between these tissues was primarily due to the extent and/or types of N-linked glycosylation (Figure S2). Differences in the major PrP^C^ polypeptides between olfactory bulb and olfactory mucosa lysates were also observed using anti-PrP monoclonal antibody SAF-32 (Figure S1). Our findings are consistent with a previous study that described a distinct PrP^C^ glycosylation pattern in extracts of the murine nasal mucosa [30]. These findings demonstrate high levels of PrP^C^ in the olfactory bulb and also a distinct PrP^C^ polypeptide pattern in the olfactory mucosa compared to the olfactory bulb and brain.

To directly test the susceptibility of the olfactory system to prion infection, Syrian golden hamsters were inoculated with the HY TME agent by either the intracerebral (i.c.) or intra-olfactory bulb (i.ob.) routes. Following i.c. inoculation of 10^6.1^ i.c. median lethal doses (LD_50_) or i.ob. of 10^5.6^ i.c. LD_50_, the incubation periods were 75±3.2 days (N = 6) and 91±3.5 days (N = 6), respectively (p-value<0.001, one-sided t-test). Western blot for total PrP (Figure 1B) and proteinase K (PK)-resistant PrP^Sc^ (Figure 1C) in normal, mock-infected (lanes 1, 4, 7) and HY TME-infected hamsters following i.c. (lanes 2, 5, 8) and i.ob. (lanes 3, 6, 9) inoculation revealed higher levels of total PrP in infected tissue homogenates than in mock-infected tissues, and the presence of PK-resistant PrP^Sc^ in the cerebellum-brainstem and olfactory bulb from HY TME hamsters, but not in mock-inoculated hamster tissues. PK-resistant PrP^Sc^ was also found at lower levels in the olfactory mucosa of HY TME-infected hamsters from both the i.c. and i.ob. groups compared to the amount in the brain based on PK digestion of 100 µg of protein from each tissue, but it was not detected in olfactory mucosa of the mock-infected hamster group (Figure 1C, lanes 7 to 9). The PrP^Sc^ polypeptide pattern in the olfactory mucosa was represented by a single polypeptide at approximately 27 kDa and it did not have the typical pattern that was characteristic of the agycosylated, monoglycosylated and diglycosylated PrP^Sc^ polypeptides present in brain and olfactory bulb (these range in molecular weight from approximately 20 to 30 kilodaltons). These findings indicate that HY TME infection resulted in a high level of PrP^Sc^ deposition in the olfactory bulb, but a lower level in the olfactory mucosa where a distinct pattern of PrP^Sc^ polypeptides was found.

**Figure 2:**
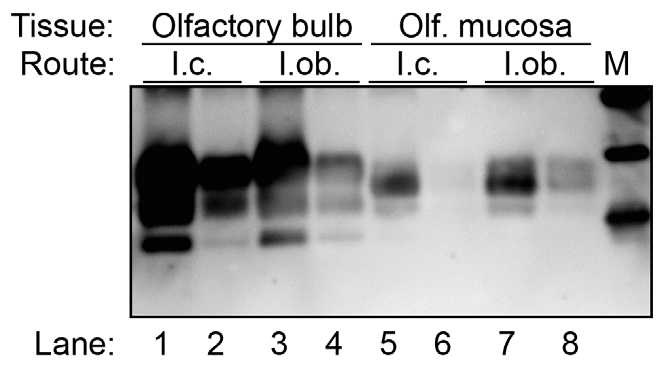
Western blot for PrP^Sc^ in olfactory bulb and olfactory mucosa following HY TME infection of hamsters. Olfactory bulb (lanes 1 to 4) and olfactory mucosa (lanes 5 to 8) lysates from clinical HY TME hamsters following intracerebral (lanes 1, 2, 5 and 6) and intra-olfactory bulb (lanes 3, 4, 7, and 8) inoculation were enriched for PrP^Sc^ by detergent extraction, ultracentrifugation, and proteinase K digestion. Western blot for prion protein was performed as described in Figure 1. Marker (M) polypeptides correspond to 20, 30, and 40 kDa.

To further define the characteristics of PrP^Sc^ in the olfactory system, PK-resistant PrP^Sc^ was enriched from lysates of the olfactory bulb and olfactory mucosa by detergent extraction and ultracentrifugation. This revealed a distinct pattern of PK-resistant PrP^Sc^ polypeptides in the olfactory mucosa extracted from nasal turbinates of HY TME infected hamsters (Figure 2). In this experiment one predominant PrP^Sc^ polypeptide was found in the olfactory mucosa extracts at approximately 26.6 kDa, while weaker intensity PrP^Sc^ polypeptides were observed at 22.2 and 28 kDa (Figure 2, lanes 5 to 8). This was distinct from the olfactory bulb where the a-, mono- and di-glycosylated PrP^Sc^ polypeptides had molecular weights of 18.4 kDa, 23.4 kDa, and 29.1 kDa (Figure 2A, lanes 1 to 4), which were similar to the PrP^Sc^ polypeptide pattern in the brain (Figure 1C). No differences in the PrP^Sc^ polypeptide pattern were observed between hamsters that were inoculated by the i.c. route (Figure 2, lanes 1, 2, 5, and 6) versus the i.ob. route (Figure 2, lanes 3, 4, 7, and 8). These findings suggest that the processing and/or formation of PrP^Sc^ in the olfactory mucosa was distinct from the olfactory bulb and brain.

### Prion infection of olfactory receptor neurons

**Figure 3:**
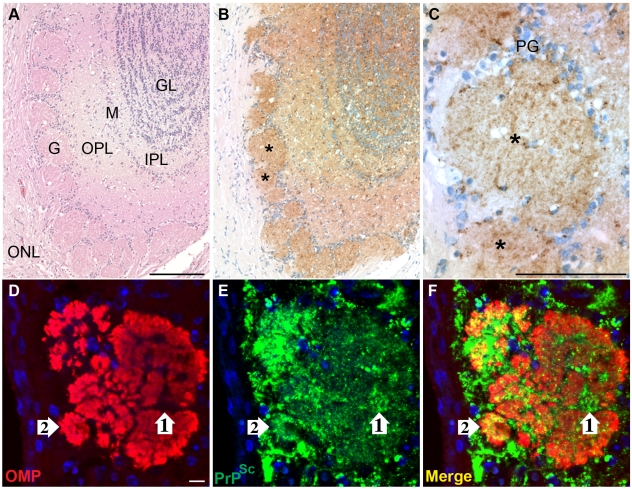
Distribution of PrP^Sc^ in main olfactory bulb following HY TME infection. Hematoxylin and eosin staining of olfactory bulb (*A*) and PrP^Sc^ immunohistochemistry (*B*, brown chromagen and hematoxylin counterstain of nuclei) in adjacent tissue section revealed a strong PrP^Sc^ pattern throughout the olfactory bulb including the granule layer (GL), inner plexiform layer (IPL), mitral cell layer (M), outer plexiform layer (OPL), and glomeruli (G), but not in the outer nerve layer (ONL). Asteriks in panel *B* illustrate PrP^Sc^ deposition in a single glomerulus, which is enlarged in panel *C* (PG = periglomerular cells). In *D* to *F*, double immunofluorescence and laser scanning confocal microscopy was used to demonstrate the relationship of olfactory marker protein (OMP)(*D*) and PrP^Sc^ (*E*) in a single glomerulus of the olfactory bulb. OMP and PrP^Sc^ immunofluorescence are merged in panel *F* and nuclei were stained with ToPro-3 (blue). White arrow #1 illustrates PrP^Sc^ immunofluorescence that was surrounded by OMP immunofluorescence in the glomerulus, while white arrow #2 is an example of PrP^Sc^ and OMP overlap in the glomerulus. Immunofluorescence staining and LSCM were performed as described in materials and methods. Scale bar is 100 µm in *A* and *C* and 10 µm in *D*.

To investigate the sites of HY TME infection in the olfactory system, immunohistochemistry (IHC) was performed on the olfactory bulb and nasal cavity in hamsters following i.ob. inoculation of the HY TME agent. In clinical hamsters, there was a strong PrP^Sc^ deposition pattern in the main olfactory bulb, most prominently in the mitral and granule cell layers, external and internal plexiform layers and glomerular layer (Figure 3A, 3B and 3C), but immunostaining was not observed in the olfactory bulb of normal, mock-infected hamsters (Figure S3A). There was a notable absence of PrP^Sc^ deposition in the outer nerve layer, which contains the axons of the ORNs whose cell bodies are located in the olfactory sensory epithelium (OSE) of the olfactory mucosa. The glomeruli of the olfactory bulb had a prominent PrP^Sc^ deposition pattern and were of particular interest since their synaptic core is the site of convergence of the axons of the ORNs with nerve terminals from the mitral and tufted cells in the olfactory bulb. Laser scanning confocal microscopy (LSCM) was used to investigate PrP^Sc^ deposition in the nerve terminals and axons of ORNs in the glomerular layer. Dual immunofluorescence for OMP and PrP^Sc^ revealed that the core of the glomeruli had widespread staining for OMP as well as PrP^Sc^ (Figure 3D to 3F). There were areas of PrP^Sc^ deposition that appeared to overlap with OMP (Figure 3, white arrow #2), but also regions of the glomerular core that were OMP-negative and were strongly PrP^Sc^-positive (Figure 3, white arrow #1). These findings suggest that the distribution of PrP^Sc^ is consistent with the nerve terminals of both the OMP-positive ORNs and OMP-negative mitral and tufted cells as they converge in the synaptic-rich glomerular layer.

**Figure 4:**
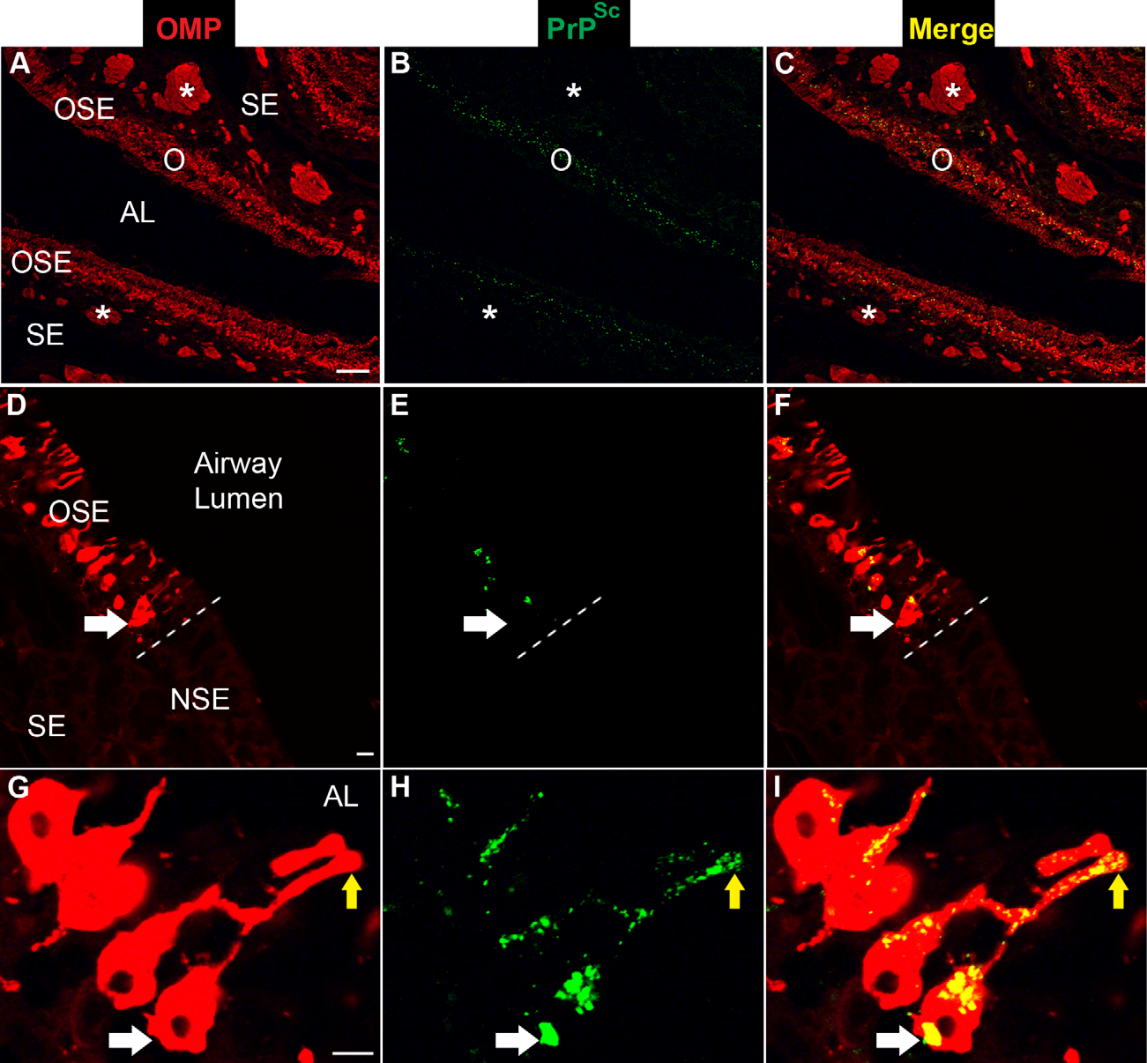
Distribution of PrP^Sc^ in olfactory sensory epithelium following intra-olfactory bulb inoculation of the HY TME agent. Laser scanning confocal microscopy of olfactory marker protein (OMP)(*A*, *D*, *G*), PrP^Sc^ (*B*, *E*, *H*), and for both OMP and PrP^Sc^ (Merge)(*C*, *F*, *I*). Panels *A* through *C*, *D* through *F*, and *G* through *I* are the same field of view. In *A* to *C*, the olfactory sensory epithelium (OSE or O) and nerve bundles (*) in the subepithelial layer (SE) express high levels of OMP and the airway lumen (AL) is observed between two opposing OSEs in the nasal turbinate. In *D* to *F*, the OSE is distinct from the nonsensory epithelium (NSE) by the presence of OMP and is separated by a dashed white line at the border between the two epithelial layers. The soma of an olfactory receptor neuron (ORN) in the OSE is identified by a white arrow and PrP^Sc^ was only observed in OMP-positive ORNs and not in the NSE. In *G* to *I*, the white arrow points to the soma of an OMP-positive ORN and the yellow arrow indicates the apical dendrite of an OMP-positive ORN, which sits adjacent to the airway lumen (*G*). PrP^Sc^ was present in both the soma and dendrites of ORNs (*H*, *I*). Immunofluorescence staining and LSCM were performed as described above. Scale bar is 100 µm in *A* and 10 µm in *D* and *G*.

To investigate the spread of HY TME infection into the nasal cavity following i.ob. inoculation, LSCM was used to localize PrP^Sc^ deposition in the nasal cavity. Dual immunofluorescence for OMP and PrP^Sc^ revealed a widespread OMP distribution in both the OSE and the nerve bundles located in the subepithelial layer (Figure 4A and 4C). These OMP-positive axons project from the ORNs in the OSE to the glomerular layer in the olfactory bulb. PrP^Sc^ deposition could be found in the OMP-positive OSE, but was not evident in the nerve bundles (Figure 4B and 4C). At higher magnification, a clear delineation was found at the transition between the OMP-positive OSE and the OMP-negative non-sensory, or respiratory, epithelium in the nasal cavity (Figure 4D and 4F). PrP^Sc^ was localized to soma and dendrites of ORNs that were OMP-positive, but not to cellular structures in the non-sensory epithelium (Figure 4E to 4F). LSCM for OMP and PrP^Sc^ in mock-infected hamster also revealed a clear delineation in OMP immunofluorescence between OSE and non-sensory epithelium, but PrP^Sc^ deposition was not observed in nasal mucosa of normal hamsters (Figure S3B and S3C). At higher magnification of ORNs, distinct PrP^Sc^ deposits were visible in the cell bodies, dendrites, and apical dendrites of individual OMP-positive cells (Figure 4G to 4I). A three-dimensional reconstruction of an apical dendritic knob revealed PrP^Sc^ deposition within this structure (Video S1), but PrP^Sc^ deposition was not observed in the dendritic knobs of ORNs from normal, mock-infected hamsters in the nasal cavity (Video S2) or in other locations in the subepithelial layer of HY TME infected hamsters including the Bowman's gland, which secretes mucus into the nasal passages. It was occasionally observed in the nasal-associated lymphoid tissue as previously reported [23].

To determine the spatial relationship between OMP and PrP^Sc^ in the ORNs, a stack of optical slices of 0.25 um thickness through the tissue were obtained by LSCM. Deconvolution of LSCM images and co-localization analysis indicated that there was a statistical difference in PrP^Sc^ immunofluorescence in OMP-positive cells between mock-infected and HY TME-infected hamsters (see average overlap coefficient in Table 1). The Manders Overlap Coefficient, M2, was calculated in order to determine the percentage of PrP^Sc^ immunofluorescence that co-localized with the OMP signal in a selected region. The M2 was determined for three regions of the OSE including the soma, dendrites, and apical dendrites of ORNs. For each portion of the ORN there was greater than a 90% co-localization of the PrP^Sc^ signal with OMP (M2>0.90, Table 1). Although PrP^Sc^ deposition in the axons of the olfactory mucosa was not readily evident by IHC or immunofluorescence at lower magnification, deconvolution and co-localization analysis of LSCM images revealed that the average overlap coefficient for PrP^Sc^ and OMP was statistically significant between mock-infected and HY TME-infected hamsters (Table 1). The M2 coefficient indicated that greater than 80% of the PrP^Sc^ signal co-localized with OMP immunofluorescence in nerve bundles. These findings indicate that HY TME infection is prominent in the soma and dendrites of ORNs and less evident in axons in the subepithelial layer of the sensory mucosa following i.ob. inoculation.

**Table 1:** Colocalization of PrP^Sc^ and olfactory marker protein in olfactory receptor neurons.

ORN Region^a^	Mock^b^	HY TME^b^	M2 values^c^
Cell body	0.003±0.002	0.152±0.009*	0.99
Dendrite	0.008±0.004	0.264±0.028*	0.99
Dendritic knob	0	0.281±0.024*	0.93
Nerve bundle	0.016±0.009	0.045±0.005*	0.83

a The individual regions of olfactory receptor neurons (ORN) was determined following anti-OMP immunofluorescence and confocal images were cropped to each region of interest prior to deconvolution and co-localization analysis as described in the materials and methods.

b Average Overlap Coefficient after Manders ± SEM, N = 27.

c Colocalization coefficient M2 indicates the percentage of PrP^Sc^ that overlaps with OMP in the region of analysis.

*p-value<0.0001, comparison between mock and HY TME infected tissues using Welch's two-sample *t*-test.

**Figure 5:**
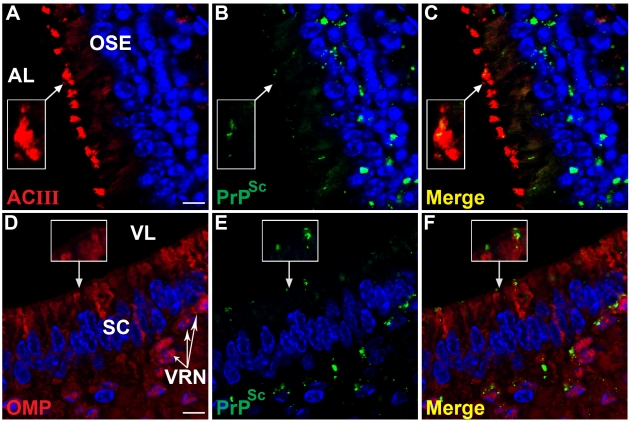
PrP^Sc^ distribution at the border of airway lumen of the olfactory and vomeronasal sensory epithelium following intra-olfactory bulb inoculation of the HY TME agent. Laser scanning confocal microscopy of adenylyl cyclase III (ACIII)(*A*), olfactory marker protein (OMP)(*D*), PrP^Sc^ (*B*, *E*), and for both ACIII or OMP and PrP^Sc^ (Merge)(*C*, *F*). Panels *A* through *C*, and *D* through *F* are the same field of view. PrP^Sc^ was prominent in the OSE including at the border with airway lumen (AL)(*B*) where it appears to overlap with ACIII (*A*), which is located on the sensory cilia that project from the terminal dendrites of ORNs into the AL (*C*). Boxed area is an inset corresponding to enlarged region indicated by arrow. In *D* to *F*, the soma, dendrites and dendritic knobs of vomeronasal receptor neurons (VRN, white arrows) in the vomeronasal sensory epithelium were OMP-positive. PrP^Sc^ was found associated with VRNs in the cell layer and at the border with the vomeronasal airway lumen (VL)(*B*). Inset illustrates enlarged area of PrP^Sc^ deposition in a location consistent with the terminal dendrites of OMP-positive dendritic knobs (*C*). PrP^Sc^ deposition was less frequently observed in the proximal dendrites as they transverse the support cells (sc, can visualize their elongated blue nuclei). Nuclei were stained with To-Pro 3. Scale bar is 10 µm.

To determine the spatial relationship of HY TME infection to the nasal airway, LSCM was performed to investigate PrP^Sc^ deposition in sensory cilia that project from the apical dendrites of ORNs into the lumen of the airway. For these studies, an antibody to adenylyl cyclase III (ACIII) was used since this membrane protein is located on the cytoplasmic side of the plasma membrane in the sensory cilia. Dual immunofluorescence staining of the OSE revealed prominent PrP^Sc^ deposition in the ORN cell layer and a more sporadic deposition pattern in the location of the apical dendrites of the ORNs (Figure 5B). ACIII immunofluorescence revealed a spatial pattern consistent with the border between the dendritic knob and lumen of the nasal cavity (Figure 5A) and when PrP^Sc^ and ACIII distributions were merged, the PrP^Sc^ pattern was consistent with deposition in ACIII-positive cilia (Figure 5C). The inset in Figure 5A to 5C illustrates a higher magnification of an ACIII-positive cilia that had evidence for PrP^Sc^ deposition. The co-localization of ACIII and PrP^Sc^ were confirmed by deconvolution of LSCM images and co-localization analysis (M2>0.90). A three-dimensional view of dual immunofluorescence for PrP^Sc^ and ACIII illustrates PrP^Sc^ deposition in the dendrites and knobs of ORNs as well as its overlap with ACIII at the tip of the ORN knobs (Video S3), but PrP^Sc^ deposition was not observed in the ACIII positive cilia in the OSE from normal, mock-infected hamsters (Figure S3D). These studies suggest that the majority of PrP^Sc^ in the region of the apical dendrites co-localize with sensory cilia and suggest that HY TME infection can be established in the cilia of ORNs.

The vomeronasal organ (VNO), which is a chemoreceptive structure at the base of the nasal septum, was another sensory structure that had evidence of HY TME infection following i.ob. inoculation. Like the olfactory epithelium, the VNO epithelium has both a sensory and non-sensory epithelium, but PrP^Sc^ deposition was only detected in the sensory epithelium of the VNO. LSCM revealed that PrP^Sc^ deposition occurred in the OMP-positive soma and apical dendrites of vomeronasal receptor neurons (VRNs), but less frequently to the proximal dendrites in VRNs (Figure 5D to 5F). The inset indicates a higher magnification of OMP-positive apical dendrites and PrP^Sc^ deposition can be observed both overlapping with OMP and also extending beyond the OMP region, perhaps in the sensory microvilli that extend from the VRN dendritic knobs into the lumen of the vomeronasal cavity. The pattern of PrP^Sc^ immunostaining in the vomeronasal sensory epithelium was similar to that in the OSE where it appeared limited to the soma and apical dendrites of sensory neurons and not to subepithelial structures.

### Prion infectivity in olfactory bulb and mucosa

To investigate the amount of HY TME infection in the olfactory mucosa following i.c. and i.ob. inoculation, TME infectivity in lysates from the olfactory bulb and olfactory mucosa extracted from nasal turbinates were measured using a hamster bioassay. The incubation period (mean ± SEM) for the four olfactory bulb lysates (68±2.3 days) was shorter (p<0.001, Tukey-Kramer analysis) than the olfactory mucosa lysates (84±1.2 days)(Table 2). However, a comparison of the incubation period for the olfactory bulb and mucosa lysates within a single hamster revealed a difference for only two of the four hamsters (p<0.001, Tukey-Kramer analysis)(Table 2, hamster i.c. #1 and hamster i.ob. #3). A comparison of the i.c., and i.ob. routes of inoculation revealed a difference in the incubation periods for the olfactory bulb lysates (p<0.001, Tukey-Kramer analysis). The lysates from the i.ob. route had a shorter incubation period compared to the i.c. route (63±1.7 days vs. 74±3.5 days). However, the route of inoculation did not effect the time to onset of HY TME following bioassay of the olfactory mucosa lysates (82±1.7 days vs. 86±1.4 days), suggesting that the greater replication in the olfactory bulb following i.ob. inoculation did not result in more TME replication in the olfactory mucosa compared to the i.c. route.

**Table 2:** Prion infectivity of olfactory bulb and olfactory mucosa lysates following HY TME inoculation.

Tissue & Hamster	Route of inoculation, intra-a	Incubation period, days ± SEM	Estimated HY TME titer, LD_50_ per microgram protein^b^	HY TME infectivity, Total median lethal doses^c^
Olfactory bulb #1	cerebral	70±4.7^d^	10^6.1^	10^9.8^
Olfactory mucosa #1	cerebral	85±2.3^e^	10^3.8^	10^8.1^
Olfactory bulb #2	cerebral	77±4.9	10^4.9^	10^9.0^
Olfactory mucosa #2	cerebral	86±1.8	10^3.7^	10^7.7^
Olfactory bulb #3	olfactory bulb	61±0.6^d^	10^8.1^	10^11.7^
Olfactory mucosa #3	olfactory bulb	85±2.3^e^	10^3.8^	10^8.2^
Olfactory bulb #4	olfactory bulb	66±3.1	10^6.9^	10^10.7^
Olfactory mucosa #4	olfactory bulb	79±1.8	10^4.6^	10^9.2^

a Following inoculation by either route, olfactory bulb and mucosa were collected from hamsters with HY TME, lysates prepared, and 25 µg protein lysate was i.c. inoculated into each recipient hamster (N = 4) in the animal bioassay.

b Infectivity titers were estimated using the incubation interval assay [29], [32], [33].

c Total median lethal doses were calculated by multiplying the HY TME LD_50_ per µg protein by the total amount of protein in each lysate.

d, e p-value<0.001 between olfactory bulb and olfactory mucosa from individual hamster, Tukey-Kramer pair-wise analysis.

For the hamster infectivity bioassay, the time interval to the onset of clinical signs of HY TME is inversely proportional to the prion titer and this enables calculation of the HY TME titer in the olfactory bulb and mucosa lysates using the incubation interval assay [31]–[33]. These incubation periods correlate to a HY TME titer of 10^4.9^ to 10^8.1^ median lethal doses (LD_50_) per ug protein for the olfactory bulb and 10^3.7^ to 10^4.6^ LD_50_/ug for the olfactory mucosa, respectively (Table 2). Although the HY TME titer of individual olfactory bulb lysates ranged from 10^2.2^ to 10^4.3^ LD_50_ greater than olfactory mucosa lysates from the same hamsters, the total amount of TME infectivity for the olfactory mucosa lysates (10^7.7^ to 10^9.2^ LD_50_) was within 100-fold of the olfactory bulb lysates (10^9.0^ to 10^10.7^ LD_50_) in three of four hamsters. In hamster #3 the total amount of TME in the olfactory bulb infectivity was 10^3.5^ LD_50_ higher than olfactory mucosa lysates (Table 2). These findings suggest that the olfactory mucosa contains very high levels of HY TME infection that are only slightly lower than those found in the olfactory bulb in most cases.

### Prion infectivity in nasal lavages

Since high levels of HY TME infectivity were found in both the olfactory bulb and olfactory mucosa following i.c. and i.ob. inoculation of hamsters, we investigated the release of prions into nasal secretions. Nasal lavages were examined in hamsters with clinical HY TME from five i.c. inoculated (incubation periods ranging from 71 to 136 days) and five i.ob. inoculated (incubation periods ranging from 90 to 107 days) hamsters (Table 3). At the terminal stages of HY TME infection, nasal lavages were collected and prion infectivity in each lavage was measured by hamster infectivity bioassay. The nasal lavages from the five HY TME hamsters infected by the i.c. route had a mean incubation period that ranged from 108±2.6 days to 252 days and overall, 22 of the 30 hamsters inoculated with these nasal lavages developed clinical TME in the hamster bioassay (Table 3). For the nasal lavages from the i.ob. inoculated group, the mean incubation period ranged from 126±9.0 days to 184±10.0 days, and 29 of 30 hamsters developed clinical disease. A comparison of the incubation periods of nasal lavage samples from i.c. versus i.ob. inoculated hamsters did not reveal a difference, 148±10.4 versus 153±8.8 days. Using the incubation interval assay to estimate the HY TME titer, the nasal lavage with the highest infectivity had an incubation period of 108±2.6 days (Table 3), which would correspond to a titer of approximately 10^3.9^ LD_50_ per ml. Since there was a 100-to-500-fold dilution of nasal fluid upon collection of nasal lavages by irrigating with one ml of PBS, it is expected that the titer of undiluted nasal fluid would be proportionally greater, or as high as 10^5.5^ LD_50_ per ml. The nasal lavage with the least amount of prion infectivity would correspond to a HY TME titer of approximately less than one LD_50_/ml, but a more precise estimate of titer will require an endpoint dilution analysis since the length of these latter incubation periods was beyond the linear range of the TME dose-response curve. The nasal lavages from the age-matched, mock-infected hamsters did not cause clinical symptoms in recipient hamsters by 255 days postinoculation (Table 3). These findings strongly suggest that HY TME infectivity is released from the OSE possibly via ORNs and subsequently shed into the lumen of the nasal airway.

**Table 3:** Prion infectivity in nasal lavages of HY TME infected hamsters.

Infection Type & Inoculation Route	Animal number	Incubation period, days	Nasal Lavage Bioassay^a^ Incubation period, days ± SEM	Affected/Inoculated
HY TME, Intracerebral				
	HY TME i.c. #1	71	165±21	5/6
	HY TME i.c. #2	73	108±2.6	6/6
	HY TME i.c. #3	129	196±20	4/6
	HY TME i.c. #4	131	124±5.8	6/6
	HY TME i.c. #5	136	252	1/6
HY TME, Intra-olfactory bulb				
	HY TME i.ob. #1	90	179±13.8	6/6
	HY TME i.ob. #2	92	126±9.0	6/6
	HY TME i.ob. #3	92	128±7.0	6/6
	HY TME i.ob. #4	93	184±10	5/6
	HY TME i.ob. #5	107	151±21	6/6
Mock, Intra-olfactory bulb				
	Mock #1	age-match control	>255	0/3
	Mock #2	age-match control	>255	0/3
	Mock #3	age-match control	>255	0/3
	Mock #4	age-match control	>255	0/3

a Nasal lavages were collected from age-matched or clinical hamsters with HY TME. For bioassay, 50 µl of nasal lavage was inoculated into 3 to 6 hamsters per sample and the duration until onset of clinical symptoms was recorded as mean ± standard error of the mean (SEM).

**Figure 6:**
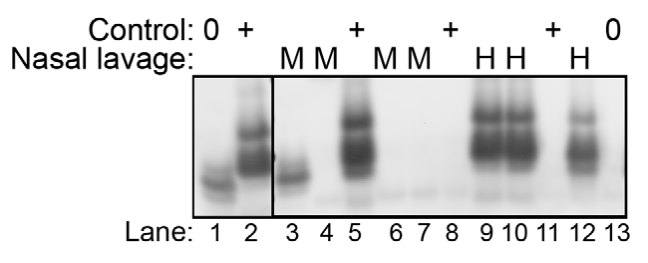
Western blot detection of HY TME infection in nasal lavages using the QuIC assay. Nasal lavages (2 µl) from mock- (M)(lanes 3, 4, 6, and 7 correspond to the four mock-infected samples from Table 3) and HY TME (H) -infected hamsters (lanes 9, 10, and 12 correspond to HY TME i.ob. #1 to #3 in Table 3) were subject to two serial rounds of the QuIC assay in order to detect low levels of PrP^Sc^. PK-resistant PrP polypeptides between 11 and 17 kDa were observed in the HY TME nasal lavage samples indicating amplification of PrP^Sc^. In mock-infected nasal lavage samples either no polypeptides or occassionally lower molecular weight polypeptides (≤12 kDa) were observed, but the latter were not observed in prion-positive control samples. Controls include mock-infected hamster brain homogenates at a 10^−2^ and 10^−6^ dilution (lanes 1 and 13, respectively), HY TME hamster brain at a 10^−6^, 10^−8^, and 10^−10^ dilution (lanes 5, 8, and 11, respectively), and 100 femtogram of purified PrP^Sc^ from 263K scrapie infected hamster brain (lane 2).

To examine nasal lavages for HY TME infectivity using a more rapid method, samples were analyzed using an in vitro PrP^Sc^ amplification method called the Quake Induced Conversion (QuIC) assay [34], [35]. This assay allows the amplification of femtogram amounts of PrP^Sc^ in hamster, sheep and human brain homogenates. During this reaction PrP^Sc^ induces the conversion of *E. coli* derived hamster recombinant PrP^C^ into a PK-resistant PrP characterized by a distinct polypeptide pattern. An aliquot of the same nasal lavage samples obtained from age-matched and HY TME hamsters used in the animal bioassay experiments (Table 3) was tested by two consecutive rounds of the QuIC assay (Figure 6). Western blot analysis revealed that the nasal lavages from HY TME infected hamsters, which had mean incubation periods between 126 and 179 days, resulted in PK-resistant PrP polypeptides of 11 to 13 kDa and 17 kDa (Figure 6, lanes 9, 10, and 12), while the nasal lavages from mock-infected hamsters did not (Figure 6, lanes 4, 6, and 7). These PK-resistant polypeptides are used to discriminate reactions seeded with PrP^Sc^ from those only containing normal brain homogenates [34], [35]. In some instances, a mock-infected brain control sample lacking PrP^Sc^ does produce only the lower molecular weight PK-resistant PrP polypeptides, which have a slightly lower molecular weight and different intensity distribution compared to the HY TME samples (Figure 6, lane 1 versus lane 2); these lower molecular weight polypeptides are sometimes observed in PrP^Sc^ -negative samples (Figure 6, lane 3) and are not considered to represent a positive result in the QuIC assay [34], [35]. In contrast, 100 femtograms of purified PrP^Sc^ resulted in a positive amplification of PrP^Sc^ (Figure 6, lane 2). Another control group included 2 µl from a 10^−6^ dilution of a HY TME-infected brain, which also resulted in the PK-resistant polypeptide pattern in the QuIC assay (Figure 6, lane 5). This dilution of hamster brain corresponds to approximately six LD_50_ of HY TME agent, while additional reactions from 10^−8^ and 10^−10^ dilutions of HY TME brain did not result in PK-resistant PrP conversion product (Figure 6, lanes 8 and 11). These results were confirmed in two additional trials of the QuIC assay. These findings indicate that a two-round QuIC reaction was effective for detecting HY TME PrP^Sc^ in nasal lavage samples containing low levels of TME infectivity.

## Discussion

The major findings of this study are 1) prion infection can replicate to high levels in the olfactory epithelium; 2) prion infection in the nasal mucosa is primarily in sensory neurons and PrP^Sc^ localization to the sensory cilia of ORNs indicates that infection extends into the airway lumen; and 3) low-to-moderate levels of prion infectivity can be recovered in nasal lavages from animals with infection of the olfactory sensory epithelium. The total amount of HY TME infectivity in the nasal turbinates was at least 10^8^ LD_50_ and in most cases, was within 100-fold of the infectivity found in the entire olfactory bulb. This represents a large amount of prion replication compared to what has been described for other peripheral tissues (excluding nervous and lymphoreticular systems) [26], [27], [36]–[38] and is most likely due to the high innervation of the olfactory epithelium. The hamster OSE has an estimated 20 million ORNs, 9 million of these with dendritic knobs, which would represent the mature ORN population [39]. This compares to an estimated 330,000 mitral and tufted cells in the main olfactory bulb (MOB) of hamsters [39] and, in small rodents, there are between 5 to 7 million granule cells in the MOB [40]. So, there are likely a greater number of neurons in the OSE than the olfactory bulb and this dense concentration of potential replication sites in the olfactory epithelium could explain the high HY TME infectivity and PrP^Sc^ levels in the olfactory mucosa. These finding are consistent with the high amount of PrP^Sc^ in the medial nasal concha of scrapie-infected sheep, which was within 10-fold of the corresponding brainstem [24], and the low-to-moderate infectivity titers in the olfactory mucosa of sheep and goats with clinical scrapie [26], [27]. The reported amount of infectivity in these mucosal lysates, ∼100 to 1,000 LD_50_, was likely underestimated due to the inefficient nature of the interspecies murine bioassay used to measure scrapie infectivity [41]. Therefore, the actual titer in mucosa from sheep scrapie are likely to be closer to those found in HY TME infection of hamsters. Further support for targeting of the olfactory system in transmissible prion diseases is found in chronic wasting disease of deer in which the olfactory system is a prominent target for PrP^Sc^ deposition [42]; we have also observed PrP^Sc^ in the nasal septum of deer and elk with CWD [28]. Therefore, the olfactory epithelium appears to be a major mucosal site for prion infection in natural prion diseases and its role in prion shedding and transmission needs to be further evaluated.

Although several studies have demonstrated prion infection in ORNs, evidence suggests that these neurons are not directly infected by environmental exposure from the airway lumen. Experimental intranasal inoculation in rodents and ruminants can cause prion disease [25], [43]–[45], but the route of neuroinvasion does not appear to be by direct entry into ORNs and subsequent spread along their axons to the olfactory bulb since the initial site of PrP^Sc^ deposition in the brain is not in the olfactory bulb [25], [43]. Although prion neuroinvasion by the intranasal route can be independent of lymphoreticular system infection in some cases [44], the evidence does not support a role for ORNs as a site of entry into the nervous system. On the other hand, ORNs do appear to be a target for prion infection following infection of the brain [23] and hence, infection is likely due to centrifugal spread of the prion agent along the olfactory and vomeronasal cranial nerves to the ORNs and VRNs, respectively [23]. This conclusion is supported in the current study in which there was widespread PrP^Sc^ deposition in glomeruli in the olfactory bulb including at both the OMP-positive ORN nerve terminals and OMP-negative regions that likely contain mitral and tufted cell processes. Transynaptic spread of the HY TME agent from mitral and tufted cells to ORN terminals in the synaptic-rich glomeruli would be a very effective mechanism to establish infection throughout the OSE since the nerve terminals of the 9 million knobbed ORNs converge at one of the 3,100 glomeruli in the hamster olfactory bulb [39]. It is estimated that there are between 1,300 and 4,667 ORNs projecting to each glomerulus [39], so infection of mitral and tufted cells at a single glomeruli can in theory result in transynaptic spread of infection to a large number or ORNs. The unique neuroanatomy in the olfactory bulb could serve to rapidly and efficiently amplify prion infection from a few dozen neurons in each glomerulus to thousands of ORNs in the OSE. Establishment of prion infection in ORNs by centrifugal spread from the olfactory bulb is also consistent with infection of the olfactory system and OSE in sporadic CJD since in this type of CJD [22], PrP^Sc^ deposition appears to have a limited distribution in peripheral tissues and would not likely gain access to the OSE from a disseminated peripheral infection [46], [47]. We propose that centrifugal prion agent spread from glomeruli in the olfactory bulb can result in a logarithmic expansion of prion infection to the ORNs. This may be the only example of an amplification of prion replication sites following centrifugal spread from the central to the peripheral nervous system. An alternate mechanism for prion infection of olfactory nerves has been proposed based on PrP^Sc^ deposition in the perineurium of the olfactory nerve bundles in scrapie-infected sheep [24]. This study suggested that spread of the scrapie agent in cerebrospinal fluid is consistent with the prion distribution to the meningeal layer of the olfactory nerve as well as a subependymal and perivascular PrP^Sc^ deposition pattern [24].

A long-standing, unresolved issue in the natural prion diseases is the source(s) and route(s) of prion transmission. Historically, it has been known that scrapie in sheep can be vertically transmitted and recent studies demonstrate that milk from scrapie-infected ewes can transmit disease to lambs from a scrapie-free flock [12], [13]. This likely represents an important route of prion transmission in scrapie, but horizontal routes including environmental contamination also have a role in prion transmission. A role for other bodily fluids in natural prion transmission in scrapie and CWD has not been firmly established despite evidence for prion infection in blood, saliva, urine, and feces as well as in carcasses of deceased deer [16]–[20]. Clearly, ruminants will be exposed to saliva, urine, and feces through direct contact with other hosts or indirectly to a contaminated environment. However, the role for these fluids is uncertain based on the low amounts of prion infectivity that were found [19], [26], [27], [36], [48]. Assuming that dose is a major limiting factor for transmission of a prion infection as it is in almost all infections, we propose that nasal secretions are a good candidate for a source of horizontal transmission since moderate levels of prion infectivity were found in nasal lavages from hamsters with HY TME. The localization of PrP^Sc^ to sensory cilia of ORNs, which form a mat in the mucus layer of the olfactory epithelium, in this study and recently demonstrated in sCJD [49], as well as the continual turnover of mature ORNs [50], [51] strongly suggest that shedding of prions into the airway lumen could result in prion infection of nasal secretions. Disruption of the nasal epithelium in ruminants from either physical insult, subcutaneous botfly infestation, or microbial infection could all result in excessive nasal discharge in sheep or cervids with a preexisting prion infection of the nasal mucosa, and therefore, lead to an enhanced shedding of prions into nasal secretions. Evidence that cofactors could contribute to prion agent shedding from the olfactory mucosa has been demonstrated in rodents with chronic kidney inflammation that results in elevated secretion of prion infectivity in urine [48] as well as in sheep with both mastitis and scrapie in which PrP^Sc^ deposition is redirected to the inflamed mammary gland [52] and may contribute to vertical scrapie transmission through secretion of prions in milk.

Our findings that HY TME infectivity could be measured in nasal lavages by animal bioassay and PrP^Sc^ was amplified using the QuIC assay indicates that nasal lavages or swabs could be used for diagnosis of prion diseases. Biopsy of the nasal epithelium has been used for diagnosis of sporadic CJD by PrP^Sc^ immunohistochemistry [53] indicating the feasibility of prion detection in nasal tissue. Therefore, analysis of nasal swabs may be an alternate approach for diagnosis or prion diseases since it is much less invasive than a tissue biopsy and this site is readily accessible and can be swabbed multiple times. Given that the QuIC assay could amplify PK-resistant PrP polypeptides from both prion samples containing approximately six LD_50_ of HY TME infectivity and nasal lavages containing low-to-moderate levels of HY TME infectivity, evaluation of nasal lavage samples using the QuIC assay warrants further investigation to determine whether it can provide a new approach for rapid prion diagnosis.

## Materials and Methods

### Animal inoculations and tissue collection

Weanling, Syrian golden hamsters (Simonsen Laboratories, Gilroy, CA) were intracerebrally (i.c.) inoculated with 50 µl of a brain homogenate from a normal hamster (i.e. mock-infected) or a TME-infected hamster containing 10^7.5^ intracerebral median lethal dose (LD_50_) per ml of the HY TME agent as previously described [29], [31] or intra-olfactory bulb (i.ob.) inoculated with 2 µl of mock or HY TME brain homogenate containing 10^8.5^ LD_50_/ml. For i.ob. inoculations, a minor surgical procedure was performed on hamsters under general anesthesia (i.e., ketamine-xylazine mixture). After shaving fur on the snout between the eyes and a betadine scrub of the skin, a single incision of the skin was made between the eyes and the skin was retracted. The subcutaneous fascia was cut and a single hole was made by gently twisting a 28-gauge needle into the incisive bone a few millimeters to the lateral side of the midline. A 30-gauge needle was inserted into the hole and 2 µl of brain homogenate was slowly injected into the left olfactory bulb. Methaclyrate glue was used to close the incision. Following inoculation of the HY TME agent, hamsters were observed three times per week for the onset of clinical symptoms, which include hyperesthesia, tremors of the head and trunk, and ataxia. Animals were euthanized at selected time points postinoculation or in the early stages of clinical disease.

For collection of tissues for immunohistochemical and immunofluorescence analyses, hamsters were intracardially perfused with periodate-lysine-paraformaldehyde (PLP) fixative, tissues dissected (e.g., brain, olfactory bulb, and nasal cavity), and processed for embedding in paraffin wax as previously described [44], [54]. For bioassay or biochemical analysis of tissues, the brain, olfactory bulb, and nasal turbinates were dissected and stored at −80°C until use. Nasal lavages were collected following exsanguination, by exposing the trachea, placing animals in a vertical position with their feet pointed upward and the head downward, inserting a 18-gauge needle, with a polished tip, and 1 cc syringe into the trachea, and slowly displacing 1 ml of PBS into the trachea. A 1.5 ml tube was placed below the nares and approximately 0.9 ml of PBS was collected; this fraction was designated the nasal lavage.

Frozen brain, specifically the brainstem and cerebellum, and olfactory bulb were homogenized in lysis buffer (i.e., 10 mM Tris-HCl, pH 7.4, 150 mM NaCl, 1 mM EDTA, 0.5% deoxycholate, and 0.5% ipegal detergent) to 10% (wt. vol.) by mincing semi-frozen tissue with a razor blade followed by trituration with hypodermic needle tips (18-, 21-, and 25-gauge) attached to a syringe. The olfactory mucosa was removed from the nasal turbinates by incubation in digestion buffer (i.e., Hepes, pH 7.5, 10 mM EDTA) containing Liberase/Blenzyme 3 (Roche Diagnostics, Indianapolis, IN.) at 50 µg/ml for 15 min at 37°C with constant shaking. The supernatant was aspirated from the large pieces of bone and cartilage and an equal volume of lysis buffer was added and incubated for an additional 15 min at 37°C with constant shaking. The supernatant was removed and combined with the supernatant from the Liberase/Blendzyme digestion step. Complete protease inhibitor (Roche Diagnostics, Indianapolis, IN.) was added and samples stored at −80°C. Protein concentration in tissue homogenates was measured using the micro-BCA assay (Pierce Protein Research, Rockford, IL). Alternatively, olfactory bulb and olfactory mucosa were homogenized in lysis buffer using a Bullet Blender (Next Advance, Averill Park, NY) and glass or zicronium beads. Enrichment for PrP^Sc^ from lysates was also performed as previously described following addition of N-lauroylsarcosine, differential ultracentrifugation in a Beckmann Optima TL-100 (Beckman Coulter Instruments, Fullerton, CA.), and limited proteinase K digestion [31], [54].

### Animal bioassay for TME infectivity

For bioassay of olfactory bulb and olfactory mucosa, 25 µg of protein from tissue lysates were i.c. inoculated into each hamster. To measure the amount of TME infectivity in the nasal lavage, 50 µl of each sample was i.c. inoculated into four to eight Syrian hamsters and the time to onset of clinical symptoms was recorded. HY TME titer is inversely proportional to the incubation period and was estimated using the incubation interval assay as previously reported [31]–[33].

### Western blot

For analysis of PrP^C^ and total PrP (i.e., PrP^C^ and PrP^Sc^), 50 to 75 µg of protein per sample was used, while for PrP^Sc^, 100 ug of protein was digested with 10 U/ml of proteinase K (PK)(Roche Diagnostics) in 0.1 ml of lysis buffer at 37°C for 1 hr. Samples were analysed on a 12% MOPS NuPAGE gel (Invitrogen, Carlsbad, CA) and proteins were transferred to PVDF membrane and incubated with monoclonal anti-PrP 3F4 antibody (gift of V. Lawson, National Institute of Allergy and Infectious Diseases, Rocky Mountain Laboratories, Hamilton, MT) at a 1∶40,000 dilution for detection of hamster PrP. To measure for the presence of olfactory receptor neurons, 50 to 75 µg of protein per sample was analyzed by western blot using an anti-olfactory marker protein (OMP) antibody (Wako Chemicals, Richmond, VA.) at a 1∶25,000 dilution. The detection system included incubation with anti-mouse or anti-donkey IgG alkaline phosphatase conjugate (Promega Corporation, Madison, WI) at a dilution of 1∶20,000. Blots were developed using CDP-Star substrate (Applied Biosystems, Foster City, CA) and imaged with a Kodak Image Station 2000MM (Eastman Kodak Company, Rochester, NY). The molecular weight of immunoreactive polypeptides was estimated using the 1D Kodak image software and the Magic Mark protein ladder (Invitrogen) were used as standards.

### Quaking Induced Conversion Assay (QuIC) for PK-resistant PrP

For amplification of low levels of PrP^Sc^ in nasal lavages the QuIC assay was performed. The QuIC assay is a modification of a published method for amplification of PK-resistant PrP that was based on the protein-misfolding cyclic amplification method [34], [35]. Briefly, 2 µl of control and samples from HY TME-positive hamsters were incubated in 96 µL buffer containing 10 µg of full length (aa 23–231) hamster recombinant prion protein (aa 23–231) that was cloned and expressed in *E. coli*. The reaction is incubated at 50°C for 8 hours with intermittent shaking (i.e. cycles of 1 min shaking and 1 min rest) using an Eppendorf Thermomixer R set at 1500 rpm. A second round of the QuIC assay was performed by removing a 2 µl aliquot of the reaction mixture and repeating the reaction. Following the second round, samples are digested with 4 µg/ml PK at 37°C for 1 hr and analyzed by Western blot using anti-PrP R20 rabbit polyclonal antibody [34], [35]. Control samples included 100 fg of PrP^Sc^ that was purified from the brain of clinical hamsters infected with the 263K strain of scrapie as well as serial 10-fold dilutions of brain homogenate from mock and HY TME inoculated hamsters.

### PrP^Sc^ immunohistochemistry

For PrP^Sc^ analysis, olfactory bulb and skulls containing the nasal cavity were collected and PrP^Sc^ immunohistochemistry (IHC) was performed as previously described [23], [44]. Briefly, animals were intracardially perfused with PLP fixative followed by immersion fixation in PLP (5 hours for olfactory bulb and 24–36 hours for skulls containing the nasal cavity and olfactory bulb). Following immersion fixation, decalcification of skulls was performed by immersion in 10% formic acid or 0.5 M EDTA (tetrasodium salt) as previously described [23], [44]. Once bone was decalcified, the nasal cavity was cut into coronal pieces prior to processing and embedding in paraffin wax. Tissues from a minimum of three animals per group were analyzed. All tissue sections were subjected to antigen retrieval by treatment with formic acid (99% wt. vol.) for 10 minutes. Tissue sections were successively incubated with anti-PrP monoclonal 3F4 antibody overnight at 4°C, then incubated with horse anti-mouse biotinylated secondary antibody (1∶400; Vector Laboratories, Burlingame, CA) at room temperature for 30 minutes, followed by streptavidin-horseradish peroxidase (HRP) at room temperature for 20 minutes. PrP^Sc^ was visualized by localization of HRP activity with DAB+ (Dako, Carpinteria, CA). Tissue sections were counterstained with hematoxylin and cover slip mounted with Aquamount (Lerner Laboratories, Pittsburgh, PA) for viewing with a Nikon Eclipse E600 microscope. Controls for PrP^Sc^ IHC included the use of mock-infected tissues and substituting a similar concentration of murine IgG isotype control for the anti-PrP 3F4 monoclonal antibody. A minimum of 30 sections throughout the thickness of the tissue was examined for each animal.

### PrP^Sc^ dual immunofluorescence

PrP^Sc^ immunofluorescence staining was combined with immunofluorescence for olfactory marker protein (OMP) or adenylyl cyclase III (ACIII)(Santa Cruz Biotechnology, Santa Cruz, CA.) as described in Table 4. Detection of primary antibodies was performed by incubation with either horse anti-mouse IgG conjugated to biotin followed by a Alexa Fluor 488 streptavidin conjugate (Molecular Probes, Portland, OR) at a 1∶400 dilution for detecton of PrP^Sc^, and either donkey anti-goat antibody conjugated to Alexa Fluor 594 antibody (1∶800; Molecular Probes, Portland, OR) or goat anti-rabbit antibody conjugated to Alexa Fluor 568 antibody (1∶800; Molecular Probes, Portland, OR). The nuclear counterstain ToPro-3 (Molecular Probes, Portland, OR) was applied to some tissue sections at a 1∶2,000 dilution for 10 min. Mowiol mounting medium was used to cover slip tissue sections. Tissues from a minimum of three HY TME-infected animals and three mock-infected animals were examined for each staining procedure.

**Table 4:** Primary antibodies used to investigate HY TME infection in olfactory mucosa by laser scanning confocal microscopy.

Antibody	Host species	Class	Dilution	Antibody Reactivity in Nasal Cavity	Source (Location)	Reference
Olfactory marker protein (OMP)	Goat	Polyclonal	1∶750	Mature olfactory receptor neurons (ORNs) and vomeronasal receptor neurons (VRNs); soma, dendrites, axons, and terminals	Wako Chemicals (Richmond, VA)	[56]
Adenylyl cyclase III (ACIII)	Rabbit	Polyclonal	1∶1,600	Sensory cilia of mature ORNs	Santa Cruz Biotechnology (Santa Cruz, CA.)	[57]
Prion Protein	Murine	Monoclonal	1∶400	Prion protein with 3F4 epitope	Gift from Victoria Lawson	[58]

### Laser scanning confocal microscopy

Images were visualized using a Zeiss LSM 510 Meta confocal system equipped with a Zeiss Plan-Apochromat ×63/NA 1.40 and ×100/NA 1.2 oil objectives. Double immunofluorescence was imaged after excitation of Alexa Fluor 488 with an Argon laser at a wavelength of 488 nm, and excitation of Alexa Fluor 568 or 594 with a Helium/Neon laser at a wavelength of 543 nm as previously described [23]. Images were scanned sequentially to minimize crosstalk between channel and the pinhole aperture was adjusted to 1.0 airy units for both channels while controlling for identical pinhole diameters and subsequent optical slice thickness. Individual images for stacks were at either 0.10 or 0.25 micron per optical slice.

### Deconvolution of confocal images

Deconvolution was performed using Huygens Essential software (version 2.7, Scientific Volume Imaging, Hilversum, The Netherlands). Using the crop tool, the region of interest (e.g. olfactory sensory epithelium, cell body, axon, dendrite, cilia) within the image was chosen for deconvolution. A similar sized region was analyzed for each comparison. A maximum likelihood estimation algorithm was applied for deconvolution of the confocal images. All images were deconvolved with identical estimations of relative background and signal to noise ratio. Deconvolution was performed on a minimum of nine image slices per individual hamster tissue, with a total of N = 27 for each morphological area in each hamster group.

### Colocalization analysis of confocal images

After deconvolution, digital images were evaluated for colocalization using the Colocalization Analyzer tool of Huygens Essential software, which provides information about the amount of spatial overlap between structures in different data channels, as previously described [23]. Colocalization coefficients were generated by the analyzer module and included the Manders overlap coefficient (MOC) and the M2 coefficients. MOC indicates the overlap of signals in image pairs, and has a value range of 0 to 1 with a value of 0 indicating no colocalization and a value of 1 indicating that all voxels (volumetric pixels) in both channels colocalize. Additional analysis was performed on HY TME-infected tissues by division of the MOC into the M2 sub-coefficients. This coefficient was calculated for voxel ranges defined by an area of interest in the image, and are insensitive to differences in signal intensities. The coefficient M2 is used to describe the contribution of channel 2 (i.e. PrP^Sc^ immunofluorescence) to the colocalized area with channel 1 (i.e. OMP or ACIII immunofluorescence). For calculation of coefficients, a minimum of N = 27 for each region of interest was used for each comparison of mock and HY TME hamsters.

### Statistical analysis

The average MOC of the confocal images generated from HY TME-infected tissues were compared to the average MOC of images generated from mock-infected tissues to evaluate the probability (p-value) that the colocalization observed in the experimental samples was greater than would be expected by chance. Statistical comparisons of MOC were performed using Welch's two-sample *t*-test using R software (R Foundation for Statistical Computing, Vienna, Austria). M2 values for HY TME were reported only when a statistical difference in MOC was found between mock- and HY TME-infected tissues. For analysis of HY TME incubation periods, a Tukey-Kramer analysis of pair-wise differences among experimental groups was performed and p-value<0.001 was considered to be significant.

### Ethics statement

All procedures involving animals were approved by the Montana State University IACUC and were in compliance with the *Guide for the Care and Use of Laboratory Animals* [55]; these guidelines were established by the Institute of Laboratory Animal Resources and approved by the Governing Board of the U.S. National Research Council.

## Supporting Information

Figure S1 Western blot for the amount of the cellular prion protein in olfactory bulb and olfactory mucosa. Lysates from normal hamster olfactory bulb (A) and olfactory mucosa from nasal turbinates (B) were analyzed by two-fold serial dilution starting with 25 µg protein (lane 1) and ending at 0.0975 µg protein (lane 9). From this representative Western blot (anti-PrP SAF-32 antibody), it is estimated that PrP^C^ could be detected in 0.39 µg of protein from olfactory bulb lysates (lane 7) and 3.12 µg of protein from olfactory mucosa lysates (lane 4). By dilution analysis there was 8-fold more PrP^C^ in the olfactory bulb than the olfactory mucosa. This analysis was performed for three individual hamsters and yielded similar results. The distinct PrP^C^ polypeptide pattern between these two tissues is also apparent between panel A and B.
(0.37 MB TIF)

Figure S2 Western blot for cellular prion protein in olfactory bulb and olfactory mucosa following deglycosylation. Lysates from normal hamster olfactory bulb (OB) and olfactory mucosa (OM)(lanes 1 to 4) were enzymatically deglycosylated with PNGase F (lanes 5 to 8) according to the manufacturer's instructions (New England Biolabs, Beverly, MA). SDS-PAGE and Western blot with anti-PrP 3F4 monoclonal antibody was performed as described in Materials and Methods. To the left of lane 1, the bracket ([) indicates polypeptides between 33.8 and 37 kDa that were prominent in the olfactory bulb lysates (lanes 1 and 2), but not in the olfactory mucosa lysates (lanes 3 and 4). To the right of the panel, the asterik (*) indicates the major prion protein polypeptide at 27.7 kDa that was present in both olfactory bulb and olfactory mucosa lysates following PNGase F digestion (lanes 5 to 8). This finding indicates that PrP^C^ from the olfactory bulb and mucosa have a similar amino acid backbone, but that there are differences in N-linked glycosylation between these two olfactory tissues. The amount of protein per lane was 80 µg (lanes 1, 2, 7, and 8), 100 µg (lanes 3 and 4), and 12.5 µg (lanes 5 and 6). Marker polypeptides (M) correspond to 20, 30, and 40 kDa.
(0.74 MB TIF)

Figure S3 PrP^Sc^ immunostaining in olfactory bulb and olfactory mucosa of normal, mock-infected hamsters. PrP^Sc^ immunohistochemistry (A) and laser scanning confocal microscopy for olfactory marker protein (OMP)(B), PrP^Sc^ (C), or adenylyl cyclase III (ACIII) and PrP^Sc^ (D) from normal, age-matched hamsters. In A, PrP^Sc^ immunohistochemistry (brown chromagen and hematoxylin counterstain of nuclei) in the olfactory bulb indicate the absence of staining in the granule layer (GL), mitral cell layer (M), outer plexiform layer (OPL), and glomeruli (G) from normal hamsters. Panels B and C are the same field of view. In B, in the olfactory sensory epithelium (OSE) there was a high level of OMP expression (red) in the soma and dendrites of olfactory receptor neurons, but OMP expression was not found in the non-sensory epithelium (NSE). The border between the OSE and NSE is demarcated by dashed white line. In C, PrP^Sc^ immunofluorescence (green) did not reveal any reactivity; nuclei were counterstained with ToPro-3 (blue). In D, the OSE was immunostained for both ACIII (red) and PrP^Sc^ (green) and counterstained with ToPro-3 (blue). The dendritic knobs (arrow) contain strong ACIII immunofluorescence at the edge of the airway lumen (AL), but PrP^Sc^ deposition was not observed in normal hamster OSE. Scale bar in panels A, C, and D are 10, 50, and 10 microns, respectively. Immunostaining and microscopy techniques are similar to those used in Figures 3, 4, and 5, and are described in the Materials and Methods.
(2.13 MB TIF)

Video S1 Video of a three-dimensional reconstruction of a dendritic knob following immunofluorescence staining for olfactory marker protein and PrP^Sc^ in the olfactory sensory epithelium of a HY TME-infected hamster. Laser scanning confocal microscopy of olfactory marker protein (OMP)(red, top panel), PrP^Sc^ (green, middle panel), and for both OMP and PrP^Sc^ (bottom panel) in the same field of view was performed as described in Figure 4. LSCM image stacks (0.10 µM optical slice) through the thickness of the tissue were used to generate a three-dimensional reconstruction of an individual dendritic knob. This movie illustrates a 360° stereoview of the dendritic knob (OMP immunofluorescence) and PrP^Sc^ located within the dendritic knob of an olfactory receptor neuron.
(9.35 MB AVI)

Video S2 Video of a three-dimensional reconstruction of a dendritic knob following immunofluorescence staining for olfactory marker protein and PrP^Sc^ in the olfactory sensory epithelium of a normal, mock-infected hamster. Laser scanning confocal microscopy of olfactory marker protein (OMP)(red, top panel), PrP^Sc^ (green, middle panel), and for both OMP and PrP^Sc^ (bottom panel) in the same field of view was performed as described in Figure 4. LSCM image stacks (0.10 µM optical slice) through the thickness of the tissue were used to generate a three-dimensional reconstruction of an individual dendritic knob. This movie illustrates a 360° stereoview of the dendritic knob (OMP immunofluorescence), but no PrP^Sc^ immunofluorescence is evident in this structure from normal hamsters.
(7.97 MB AVI)

Video S3 Video of a three-dimensional reconstruction of a dendritic knob following PrP^Sc^ and ACIII immunofluorescence in the dendrites and cilia of olfactory receptor neurons following HY TME infection. Laser scanning confocal microscopy and immuofluorescence staining was performed for PrP^Sc^ (green) and ACIII (red) as described in Figure 5. Image stacks (0.25 µM optical slice) through the thickness of the olfactory sensory epithelium were used to generate a three-dimensional reconstruction of the dendrites and dendritic knob of olfactory receptor neurons. This movie illustrates a 360° view of the spatial relationship of PrP^Sc^ and ACIII in the dendrites and sensory cilia of olfactory receptor neurons.
(4.22 MB AVI)
