# Supplementary figures and images for: Prion Shedding from Olfactory Neurons into Nasal Secretions
Source: PLoS Pathog. 2010 Apr 15;6(4):e1000837. doi: 10.1371/journal.ppat.1000837 (PMC2855443; doi:10.1371/journal.ppat.1000837)

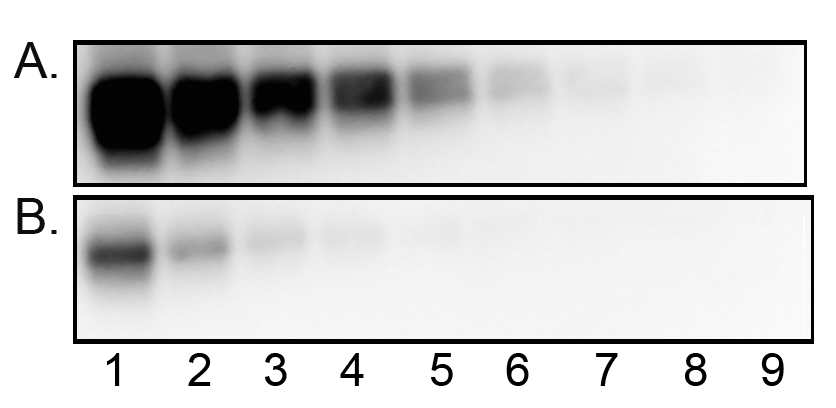

Supplement: Figure S1 — Western blot for the amount of the cellular prion protein in olfactory bulb and olfactory mucosa. Lysates from normal hamster olfactory bulb (A) and olfactory mucosa from nasal turbinates (B) were analyzed by two-fold serial dilution starting with 25 µg protein (lane 1) and ending at 0.0975 µg protein (lane 9). From this representative Western blot (anti-PrP SAF-32 antibody), it is estimated that PrPC could be detected in 0.39 µg of protein from olfactory bulb lysates (lane 7) and 3.12 µg of protein from olfactory mucosa lysates (lane 4). By dilution analysis there was 8-fold more PrPC in the olfactory bulb than the olfactory mucosa. This analysis was performed for three individual hamsters and yielded similar results. The distinct PrPC polypeptide pattern between these two tissues is also apparent between panel A and B. (0.37 MB TIF) [file ppat.1000837.s001.tif]

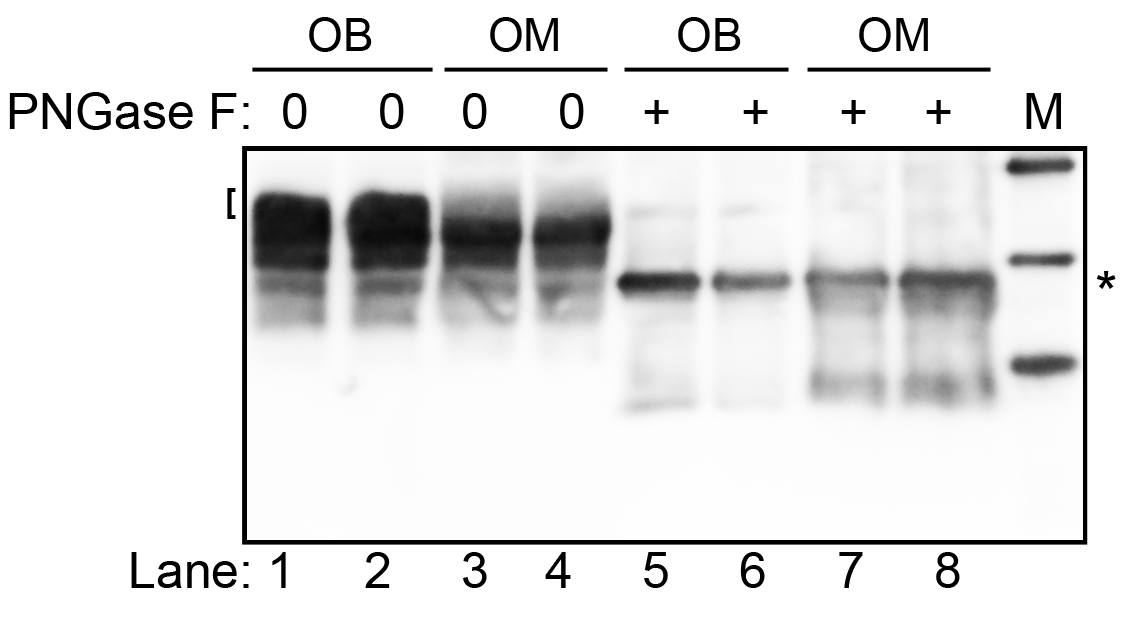

Supplement: Figure S2 — Western blot for cellular prion protein in olfactory bulb and olfactory mucosa following deglycosylation. Lysates from normal hamster olfactory bulb (OB) and olfactory mucosa (OM)(lanes 1 to 4) were enzymatically deglycosylated with PNGase F (lanes 5 to 8) according to the manufacturer's instructions (New England Biolabs, Beverly, MA). SDS-PAGE and Western blot with anti-PrP 3F4 monoclonal antibody was performed as described in Materials and Methods. To the left of lane 1, the bracket ([) indicates polypeptides between 33.8 and 37 kDa that were prominent in the olfactory bulb lysates (lanes 1 and 2), but not in the olfactory mucosa lysates (lanes 3 and 4). To the right of the panel, the asterik (*) indicates the major prion protein polypeptide at 27.7 kDa that was present in both olfactory bulb and olfactory mucosa lysates following PNGase F digestion (lanes 5 to 8). This finding indicates that PrPC from the olfactory bulb and mucosa have a similar amino acid backbone, but that there are differences in N-linked glycosylation between these two olfactory tissues. The amount of protein per lane was 80 µg (lanes 1, 2, 7, and 8), 100 µg (lanes 3 and 4), and 12.5 µg (lanes 5 and 6). Marker polypeptides (M) correspond to 20, 30, and 40 kDa. (0.74 MB TIF) [file ppat.1000837.s002.tif]

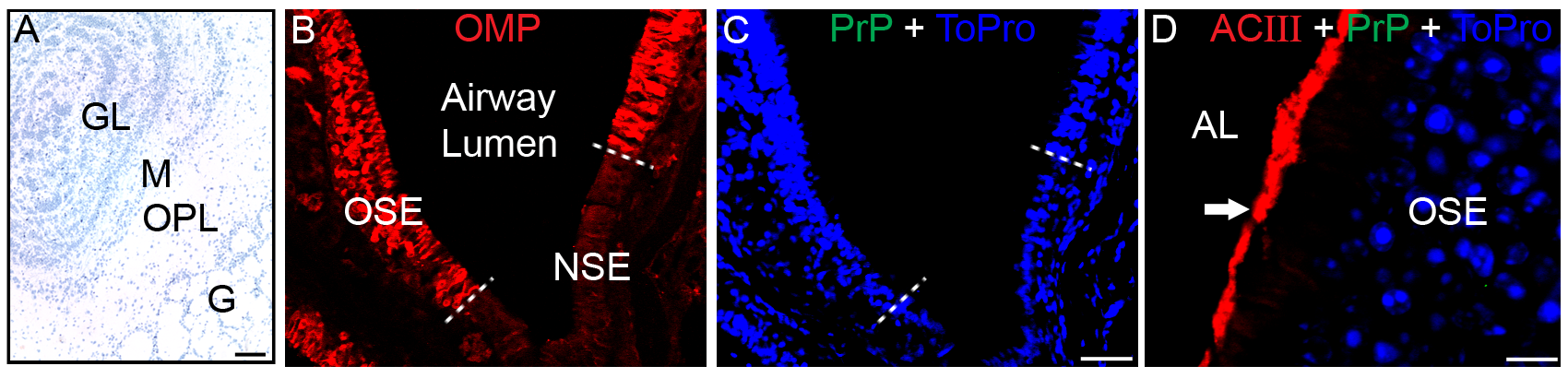

Supplement: Figure S3 — PrPSc immunostaining in olfactory bulb and olfactory mucosa of normal, mock-infected hamsters. PrPSc immunohistochemistry (A) and laser scanning confocal microscopy for olfactory marker protein (OMP)(B), PrPSc (C), or adenylyl cyclase III (ACIII) and PrPSc (D) from normal, age-matched hamsters. In A, PrPSc immunohistochemistry (brown chromagen and hematoxylin counterstain of nuclei) in the olfactory bulb indicate the absence of staining in the granule layer (GL), mitral cell layer (M), outer plexiform layer (OPL), and glomeruli (G) from normal hamsters. Panels B and C are the same field of view. In B, in the olfactory sensory epithelium (OSE) there was a high level of OMP expression (red) in the soma and dendrites of olfactory receptor neurons, but OMP expression was not found in the non-sensory epithelium (NSE). The border between the OSE and NSE is demarcated by dashed white line. In C, PrPSc immunofluorescence (green) did not reveal any reactivity; nuclei were counterstained with ToPro-3 (blue). In D, the OSE was immunostained for both ACIII (red) and PrPSc (green) and counterstained with ToPro-3 (blue). The dendritic knobs (arrow) contain strong ACIII immunofluorescence at the edge of the airway lumen (AL), but PrPSc deposition was not observed in normal hamster OSE. Scale bar in panels A, C, and D are 10, 50, and 10 microns, respectively. Immunostaining and microscopy techniques are similar to those used in Figures 3, 4, and 5, and are described in the Materials and Methods. (2.13 MB TIF) [file ppat.1000837.s003.tif]
